# An Individualized Approach to Neuroplasticity After Early Unilateral Brain Damage

**DOI:** 10.3389/fpsyt.2019.00747

**Published:** 2019-11-19

**Authors:** Katerina Gaberova, Iliyana Pacheva, Elena Timova, Anelia Petkova, Kichka Velkova, Ivan Ivanov

**Affiliations:** ^1^Department of Pediatrics, University Hospital “St.George”, Plovdiv, Bulgaria; ^2^Complex of Translational Neuroscience, Medical University - Plovdiv, Plovdiv, Bulgaria; ^3^Department of Pediatrics and Medical Genetics, Medical University - Plovdiv, Plovdiv, Bulgaria; ^4^Department of Medical imaging, Medical University - Plovdiv, Plovdiv, Bulgaria

**Keywords:** pre/perinatal brain lesion, functional MRI, motor reorganization, sensory reorganization, language reorganization, dyslexia, functional capacity, predictive factors

## Abstract

**Introduction:** Reorganization after early lesions in the developing brain has been an object of extensive scientific work, but even growing data from translational neuroscience studies in the last 20 years does not provide unified factors for prediction of type of reorganization and rehabilitation potential of patients with unilateral cerebral palsy (UCP) due to pre/perinatal insult.

**Aim:** To analyze the type of motor, language, and sensory brain reorganization in patients with right-sided cerebral palsy due to pre/perinatal isolated left-sided brain lesions taking into consideration the type (cortico-subcortical or periventricular) and extent (gray and white matter damage) of the lesion, etiology, comorbidity, and other postnatal factors that could have played a role in the complex process of brain plasticity.

**Material and Methods:** Eight patients with unilateral right cerebral palsy were included in the study. The individual data from fMRI of primary sensory, motor, and language representation were analyzed and compared with respective comprehensive etiological, clinical, and morphological data. Patients were examined clinically and psychologically, and investigated by structural and functional 3T GE scanner. A correlation between the type and extent of the lesion (involvement of cortical and subcortical structures), timing of lesion, type of reorganization (laterality index), and clinical and psychological outcome was done.

**Results:** Significant interindividual diversity was found in the patient group predominantly in the patterns of motor reorganization. Patients with small periventricular lesions have ipsilesional representation of primary motor, sensory, and word generation function. Patients with lesions involving left cortico-subcortical regions show various models of reorganization in all three modalities (ipsilesional, contralesional, and bilateral) and different clinical outcome that seem to be impossible for prediction. However, patients with UCP who demonstrate ipsilesional motor cortical activation have better motor functional capacity.

**Conclusion:** The type and size of the pre/perinatal lesion in left hemisphere could affect the natural potential of the young brain for reorganization and therefore the clinical outcome. Much larger sample and additional correlation with morphological data (volumetry, morphometry, tractography) is needed for determination of possible risk or protective factors that could play a role in the complex process of brain plasticity.

## Introduction

Translational neuroscience has developed over the past few decades as innovative field to bridge knowledge across disciplines in medicine and especially to translate data/knowledge from fundamental neurosciences (such as neurobiology) to explanation of human brain functions in health and disease (1).

Brain reorganization after early lesions in the developing brain has been an object of extensive scientific work, but even with the achievements from translational neuroscience in the last 20 years it is still not clarified which factors could predict type of reorganization and rehabilitation potential of patients with unilateral cerebral palsy (UCP) due to pre/perinatal insult. UCP implies an excellent model for studying brain plasticity. It comprises heterogenic conditions in terms of etiology, timing, morphology, clinical signs, and severity of impairments (2). Variety of unilateral brain lesions, acquired in the pre/perinatal period could lead to UCP with abnormal motor behavior as the core feature of this unprogressive condition. Lesions acquired during the first two trimesters of pregnancy interfere with the processes of neuronal migration, proliferation, and cortical organization, leading to cortical malformations and, thus, disturb the normal function of the affected area. Lesions that are acquired in the third trimester and in early postnatal life disrupt structures that are already formed, but also interfere with the processes of dendritic arborization, axonal sprouting, and myelination. The size and extent of the lesion in the hemisphere, as well as its type—periventricular lesion (PVL) or cortico-subcortical lesion (CSL)— could affect these processes in different ways (3). The brain maturation and reorganization might be additionally influenced by the factors leading to the initial insult like genetic conditions, infections, neonatal encephalopathy, and medical intervention, and also by postinsult events like early therapeutic intervention and epilepsy. Recent studies comment on the inability of the immature brain to follow the simple Kennard principle due to many events that shape different developmental trajectory (4). Taking all these reasons together, applying one unified model of brain reorganization in patients with UCP seems impossible, and three main models of reorganization—ipsilesional, contralesional, and bilateral have been widely discussed (5). Despite the growing number of studies in this area, it still remains difficult to predict individual remodeling. Functional MRI (fMRI) is a novel method, which is an excellent tool for studying brain reorganization in various brain functions (motor, sensory, language, cognition, etc.) using different tasks. Due to its noninvasive nature and good spatial and acceptable temporal resolution, it is widely used in studies of developmental disorders. UCP represents an appealing model for the study of brain plasticity for several main reasons:

- Lesion is acquired before 2 years of age, when reorganization potential of the contralesional hemisphere is significant due to the ongoing development of the normal projections (6).
- The study is conducted when reorganization is already completed (it’s most intensive during 6 months after injury) and the type of reorganization is more or less fixed (7).
- Most of the lesions could be specified in time of appearance with high certainty depending on risk factors, prenatal imaging techniques, type of lesion, etc. (3).

Unfortunately, it remains very difficult to homogenize the group of studied patients so that a large sample study can be achieved for sufficient statistical results. UCP is a rare disorder with cumulative prevalence of 0.6 to 1 *per* 1,000 live births. The exact prevalence of CP is unknown in Bulgaria, but the largest Bulgarian study on CP included 143 patients with UCP out of CP sample of 521 patients (personal correspondence with Dr. Elena Rodopska, University Hospital “St. Naum,” Sofia, Bulgaria). UCP is an umbrella term that includes several conditions that vary in etiology and morphology (2). With demographic variables added, it is almost impossible to obtain homogeneous group of patients. Moreover, the study samples are reduced due to absence of motivation or inability to perform the task inside the MRI. These challenges explain the relatively small sample number of patients with UCP that were included in fMRI studies in literature—between 3 and 25 (5).

The aim of this study is to analyze the type of motor, language, and sensory brain reorganization in patients with right-sided cerebral palsy due to pre/perinatal isolated left-sided brain lesions taking into consideration the type (cortico-subcortical or periventricular) and extent (gray and white matter damage) of the lesion, etiology, comorbidity, and other postnatal factors that could have played a role in the complex process of brain plasticity.

## Material and Methods

### Participants

The study was performed prospectively in the Complex of Translational Neuroscience, Medical University—Plovdiv for the period 2017–2019. Eight patients with diagnosis of right-sided cerebral palsy (three males, aged 13–15 years, and five females, aged 10–30 years) were included in the study.

The inclusion criteria were:

- Congenital right-sided hemiparesis with MACS level ≤3
- Mental age >7 years
- Unilateral left-sided brain lesion proved by a brain image
- Fulfilled informed consent (from either the patient or his parent if the patient is under the age of 18 years) for participation in the study

The exclusion criteria were:

- Patient and his parents’ disagreement for participation in the study
- Impossibility to stay calm during fMRI scan
- Uncooperativeness of the patients or inability to perform the fMRI paradigms
- Presence of implants in the patients’ body, which is contraindication for MRI

The patients were examined by clinical and psychological tests and investigated by structural 3T scan and fMRI tests.

The study design was approved by the Ethical Committee of Medical University—Plovdiv.

### Procedures

#### Clinical and Psychological Examination

The medical interview with the patients included questions about familial risk factors, risk factors during pregnancy, delivery, and early postnatal period, time of diagnosis, laboratory, genetic, and imaging data, type and duration of rehabilitation, and comorbidities (epilepsy, cognitive deficits, visual and other sensory deficits). Clinical investigation (physical and neurologic examination) was done by either a specialist in pediatric neurology or adult neurologist.

Severity of movement difﬁculties was evaluated by the score on the Manual Ability Classiﬁcation System (MACS) and the Modiﬁed Ashworth Scale (MAS). The MACS classiﬁes a person’s ability to handle objects in important daily activities across a ﬁve-point scale (level I—handle most objects easily; to level V—severely limited in their ability) (8). MAS further characterized the children by documenting severity of movement restriction due to spasticity across the elbow, wrist, ﬁngers, and thumb (0—indicating no movement restriction, to 4—reﬂecting rigidity/severe contracture). The sensory examination included examination of touch, pain, joint position sense, stereognosis, and graphesthesia. Rehabilitation was classified as absent (−), rare—less than one course *per* month (+), moderate—one or more courses *per* month (++), and frequent—every day (+++). Time of onset of rehabilitation was registered for every patient. All participants received psychological evaluation by a psychologist with IQ (WISC-IV), as well as evaluation for dyslexia with DDE-2 battery, both adapted in Bulgarian language (9, 10).

#### Mri Procedure

##### Data Acquisition

Scanning of all patients was executed on a 3Т MRI system—GE Discovery 750w with a protocol including a structural scan: SagT1 FSPGR BRAVO, slice thickness 1 mm, matrix 256 × 256, flip angle 12°. Additional AxFLAIR scan was performed for better qualification of lesions. The protocol for all functional scans contained 2D Echo planar imaging, slice thickness 3 mm, matrix 96 × 96, relaxation time 3,000 ms, echo time −30, and flip angle 90°. Before each functional scan, five dummy time series were acquired.

##### Experimental Paradigms

All patients were familiarized with the fMRI procedure through animated presentation. The experiment contained five paradigms, each implemented in block design: two active movement conditions—left (ML) and right (MR) hand finger tapping; two passive sensory conditions—left (SL) and right (SR) hand brushing; and a word generation paradigm. During one session, each task was performed for 30 sec and repeated 5 times after 30 sec of rest. Finger tapping task was performed with repetitive touching of first and second finger with frequency of approximately 1 Hz. Movements were directly observed by an experimenter. Hand brushing task was performed with gentle brushing of the back of each hand with frequency of 1 Hz by the same experimenter. The beginning of each active and passive block for the motor and sensory paradigm was presented on the screen in front of the patient with the word “Start” and “Stop,” respectively. For the word generation task there were five different letters presented on the screen (one for each block of the task) and the patient was asked to think of as many words as possible, starting with the letter presented on the screen (silent generation of words). The total duration of the functional scan was 25 min. Paradigms were shown in a randomized order.

##### Analysis of Imaging Data

Preprocessing steps were carried out using custom routines available in SPM12 (Wellcome Department of Imaging Neuroscience, University College, London, UK, http://www.fil.ion.ucl.ac.uk/spm) . Images were corrected for head movements by realigning all images with the first image of the first session, and a mean image of the realigned volumes was created. To remove variance due to unwanted head movements that might have been task-related, images were unwrapped (11). The 3D-dataset was segmented in native space, using a unified segmentation approach (12). The segmented tissue maps were coregistered to the mean functional image from the first session. The crucial step of normalization capitalizes on the fact that chronic lesions are overwhelmingly classified as CSF during tissue segmentation (13). This tissue class is then used as the basis for an automatically generated lesion mask which in turn is used to implement a cost-function masking approach (14) during spatial normalization. These segmentation parameters were used to normalize the functional series to a final resolution of 2×2×2 mm. In the end, the images were spatially smoothed 8 mm full width at half-maximum (FWHM).

The model for first-level analysis was then specified with parameters estimated, and t-contrasts defined for active versus passive condition for all five experiments (Motor Right—MR, Motor Left—ML, Sensory Right—SR, Sensory Left—SL, Word Generation—WG). The level of significance was set at *p* < 0.05 familywise error corrected and cluster extent threshold of 10 voxels. Statistical results were presented using SPM extension Bspm view (http://www.bobspunt.com/software/bspmview/) .

Laterality index (LI) was calculated for motor and language representation using the commercially available tool LI (http://www.medizin.unituebingen.de/kinder/en/research/neuroimaging/software/) . LI was obtained by computing LI = (nL − nR)/(nL + nR), where nL and nR are the number of activated voxels in left (LH) and right (RH) hemisphere, respectively (15). The absolute value 0.10 was used as threshold for deﬁnite lateralization (16). Patients with a positive index (LI > 0.10) were considered left-lateralized for language, while those with a negative index (LI < −0.10) were considered as right-lateralized. Values of |LI| = 0.10 represent a “bilateral” or uncertain activation. For calculation of LI for language representation, the total number of voxels in the gray matter was used, while LI for motor representation was calculated using the number of activated voxels only in the primary motor cortex (PMC).

The extent of injury of gray matter (GM) and white matter (WM) was classified according to maximum width of lesion as 1 = mild (<10 mm), 2 = moderate (10–20 mm), and 3 = severe (>20 mm) (17). Lesional volume was calculated and visualized using MRICron (https://www.nitrc.org/projects/mricron/).

Presumed timing of the lesions was judged by the criteria offered by (18) malformation of the cortical development (MCD) occurs during I and II trimester, while PVL and CSL during III trimester or perinatally. In addition, PVL could be approximately assigned to the period of 24–36 weeks of gestation, and CSL after 36 weeks of gestation (19).

##### Statistical Analysis of Behavioral and Imaging Data

Due to the small number of subjects, no statistical analysis on group level was done. Patients were divided in three main groups depending on type of the lesion, and comments were made on group and individual level, associated with various behavioral data.

### Results

#### Demographic and Clinical Data With Risk Factors for Pre/Perinatal Brain Lesions

**Figure 1:**
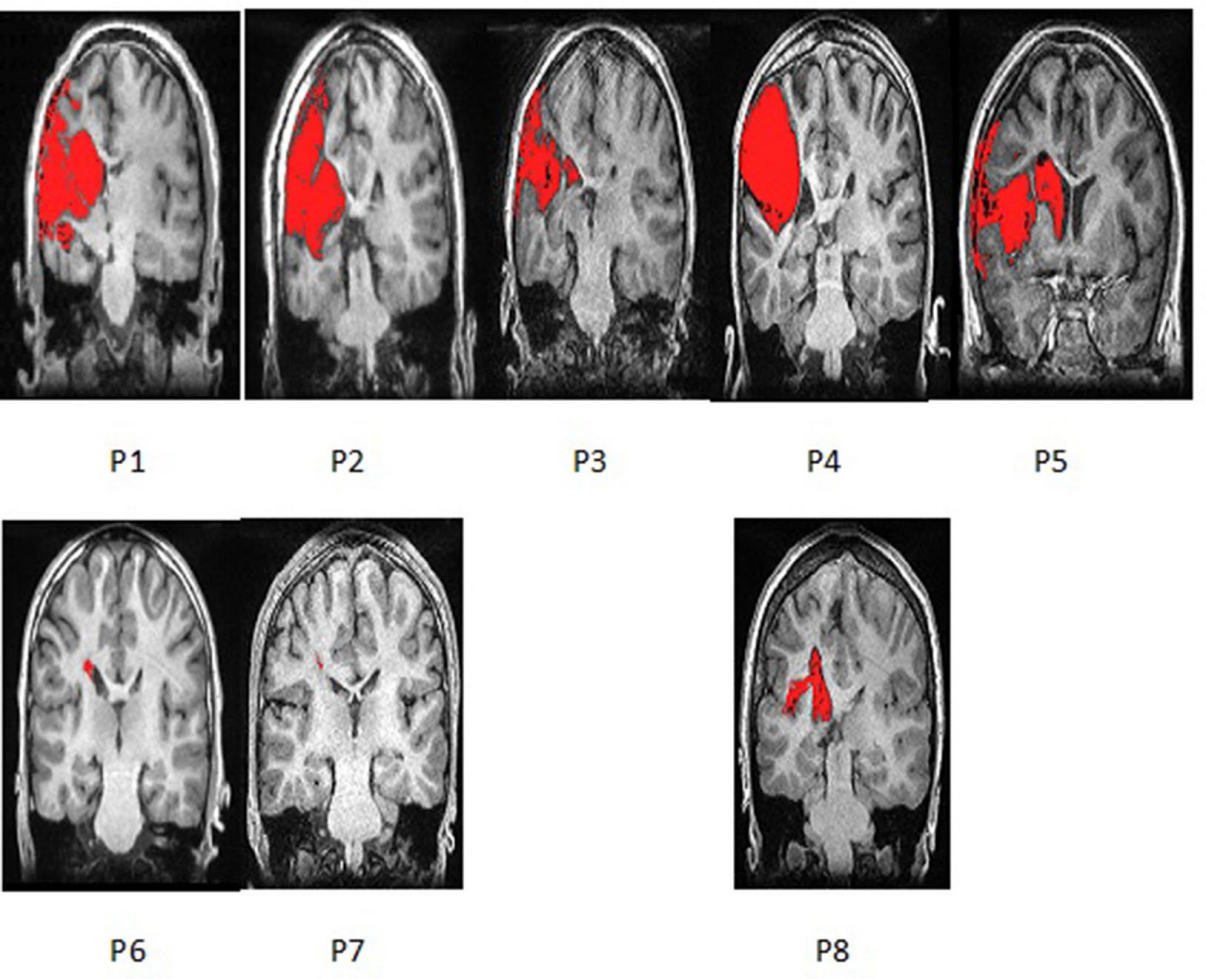
Coronal section of T1 Structural MRI of all eight patients showing their brain lesions (lesion represented in red color). Images are presented *via* MRICron and therefore flipped—left side of the brain is on the left side of the image.

Five of the patients had radiological evidence of involvement of unilateral cortical and subcortical regions in the territory supplied by the left middle cerebral artery, further referred to as CSL lesions (P1–5), probable arterial infarction. Two had no cortical involvement but only unilateral PVL, presenting with mild enlargement of the frontal horn of the left lateral ventricle and periventricular T1 hypointensity and FLAIR hyperintensity—presumable venous infarctions or unilateral periventricular leukomalacia (P6 and P7). One had MCD—closed lip left-sided schizencephaly (P8) (**Figure 1**).

All five patients with CSL (MCA-infarction) had second and third degree of involvement of both cortex and adjacent white matter tracts including partially the Rolandic and the Broca area (**Table 1**). For the patient with MCD this involvement was of lesser extent (first degree). Both patients with PVL had involvement of the anterior portion of the internal capsule, defined as first-degree white matter injury. There was no cortical involvement for the patients with PVL.

**Table 1:** Demographic and clinical data of patients.

Subject	Age	Lesion	Extent of injury - classification	Extent of injury (mm^3^)	Prenatal risk factors	Thrombophillya	Neonatal encephalopathy	Time of insult	Rehabilitation	MACS level	Sensory deficit	Epilepsy	IQ	Dyslexia
P1	26–30	LMCA infarction	3	26.72	Eclampsia	NA	No	Late III trimester	+	3	No	Yes	70	Yes
P2	16–20	LMCA infarction	3	42.59	No	NA	Yes—birth trauma	Birth	+	3	No	No	60	Yes
P3	10–15	LMCA infarction	2	16.88	No	NA	No	Late III trimester	+	2	No	Yes	80	Yes
P4	10–15	LMCA infarction	3	59.65	No	Yes	No	Late III trimester	–	1	No	Yes	89	Yes
P5	10–15	LMCA infarction	2	15.04	No	NA	Yes—cardiac arrest	Birth	+++	1	No	Yes	75	No
P6	10–15	PVL small leftlesion	1	0.73	No	NA	No	Early III trimester	++	1	No	No	98	Yes
P7	10–15	PVL small left lesion	1	1.21	Yes—eclampsia	Yes	No	Early III trimester	–	1	No	No	78	No
P8	21–25	MCDleft	1	9,18	Yes—bleeding	NA	Yes—breathing problem	II trimester	+	3	No	Yes	50	NA

F, female; M, male; LMCA, left middle cerebral artery; PVL, periventricular lesion; MCD, malformation of cortical development; NA, not available.

Functional capacity measured by MACS was very good in both patients with PVL, poor in the patient with MCD, and variable in the patients with CSL. The same distribution was found for the degree of limb spasticity, measured by MAS.

No patient was found to have sensory deficits during clinical examination of touch, pain, joint position, stereognosis, and graphesthesia.

Five of six patients with cortical involvement (four CSL and the MCD) had epilepsy with partial or secondary generalized seizures originating from the left hemisphere. All of them were under a stable dose of anticonvulsive medication by the time of the study.

IQ varied from 50 to 90 (**Table 1**).

Four out of five patients with CSL and one out of two with PVL were dyslexic. One patient couldn’t be evaluated for dyslexia, because of lack of cooperativeness.

The risk factors presumably involved in the brain damage causing the lesions were: risk pregnancy (found in three patients), prematurity (none of the tested patients), genetic thrombophilia factors (one patient), birth asphyxia and trauma (one patient). Timing of these factors was referred to occurrence of the lesion, i.e., severe birth asphyxia in P5 and finding of left porencephalic cyst leads to diagnosis of left middle cerebral artery infarction that we assumed as having occurred at birth. Maternal bleeding around 26th gestational week and finding of closed lip shizencephalic cleft could also suggest the time of insult.

#### Activation During Motor Task With the Impaired Hand

All eight patients completed finger tapping task successfully with the right hand. Mirror movements during the task were observed in P1 and P3.

**Figure 2:**
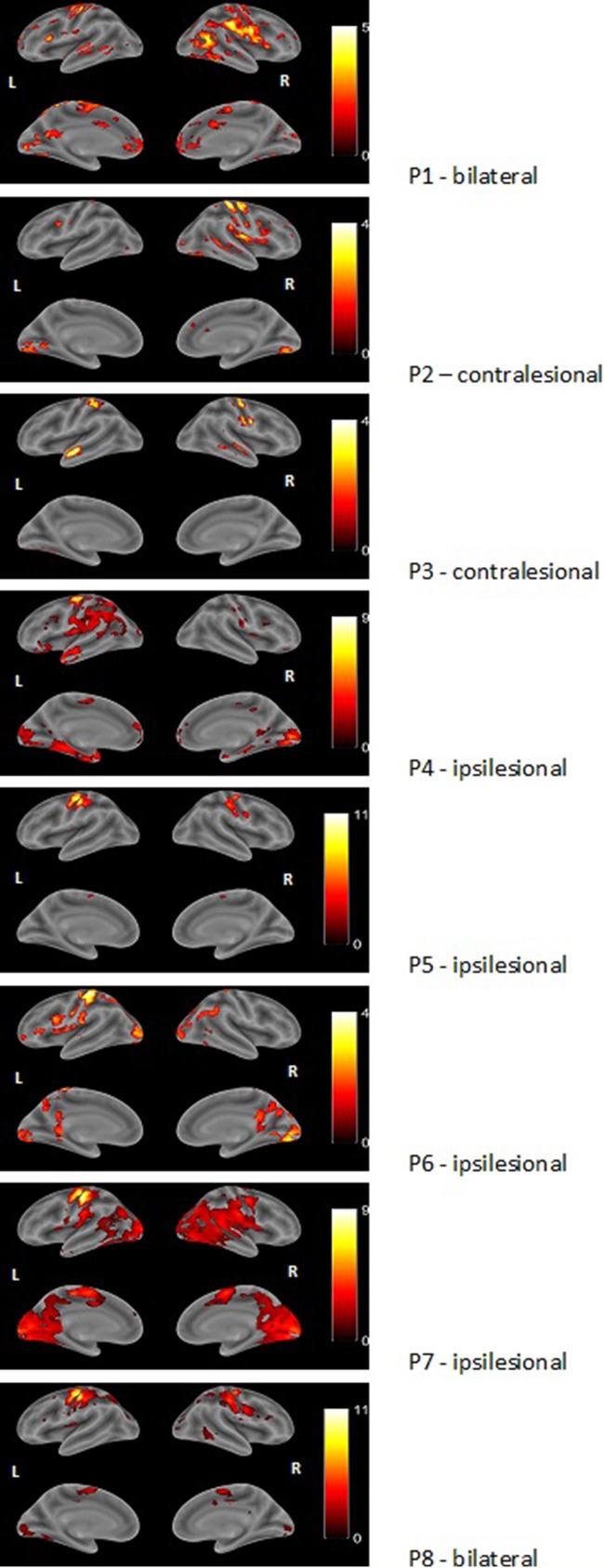
Examples of render images of the three different patterns of activation during motor task with the impaired right hand (MR task). Color bar represents t-value.

During finger tapping with the impaired hand three types of activation in the PMC were found (**Figure 2**):

1. Predominant activation in preserved areas of left precentral gyrus—M1 (ipsilateral to the lesion) in four patients (two with PVL: P6 and P7, and two with CSL: P4 and P5)
2. Bilateral distribution of the activation in PMC—in two patients (one with CSL: P1, and the one with MCD: P8)
3. Activation only in the contralesional (right) precentral gyrus—in the other two patients with CSL (P2 and P3)

Additional extensive activation in various cortical regions in both hemispheres was observed in all patients, involving areas outside typical motor system representation.

A clear association was found between functional capacity of the patients and the type of reorganization: all four patients with MACS level I showed predominantly left-sided activation in the PMC, while those with MACS level II and III had either bilateral or right-sided activation. Patients with PVL showed strong ipsilesional activation, while patients with cortical involvement had all three patterns of activation in their PMC. As to extend of the lesion, P6 and P7 who had first degree of injury and no GM involvement showed ipsilesional activation, while the other patient with small lesion (<10 mm) but with cortical involvement showed bilateral activation with LI (−0.03). Patients with second degree of injury showed two completely different patterns—contra- or ipsilesional activation (P3 and P5), similar to P1, P2, and P4, who had the greatest extend of injury to both white and gray matter. Rehabilitation frequency didn’t seem to affect the reorganization—P5 and P6 who showed ipsilesional activation had very frequent rehabilitation, unlike P 4 and P7 who had similar lesions and patterns of motor activation, but had barely conducted rehabilitation.

#### Activation During Motor Task—Nonimpaired Hand

**Figure 3:**
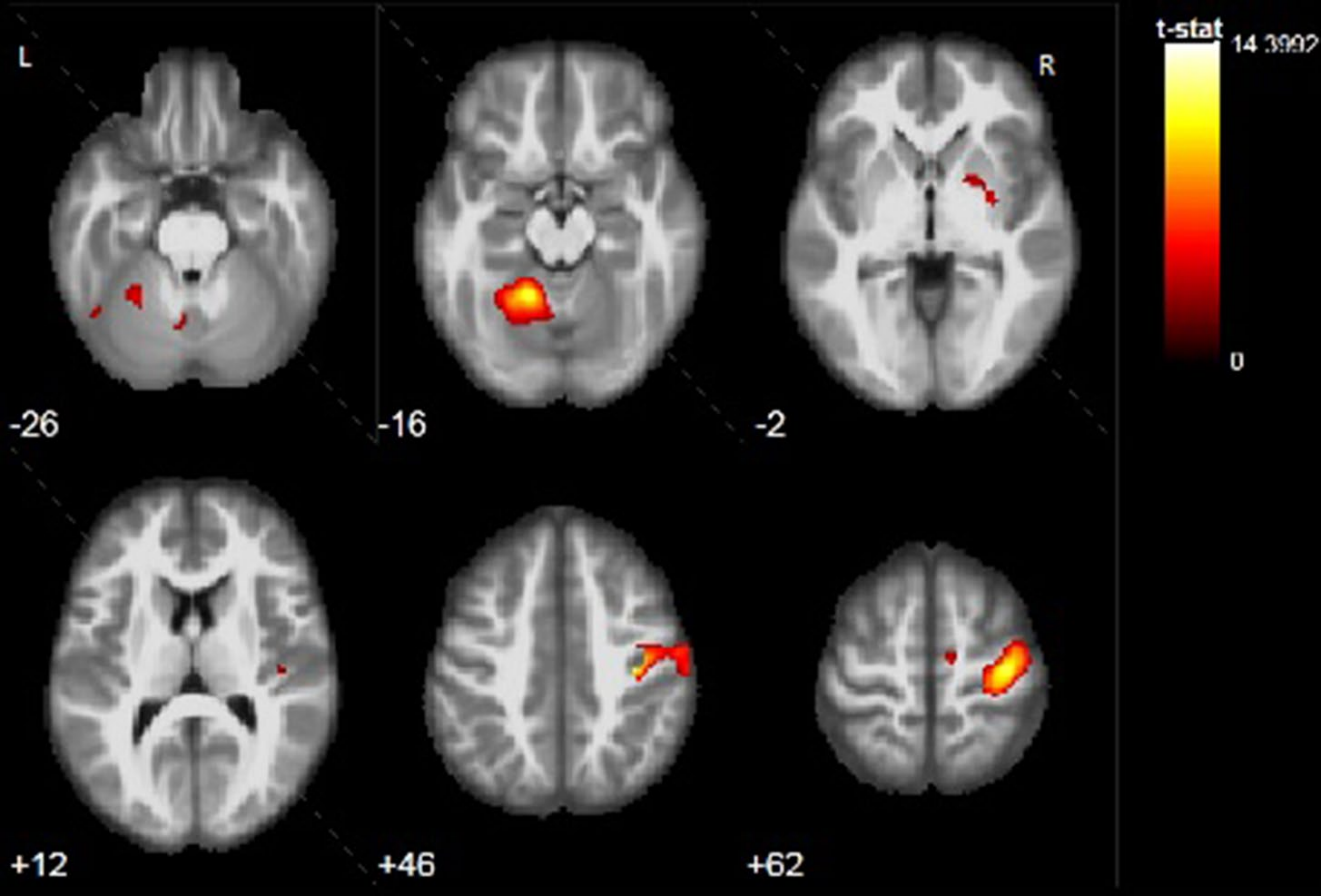
Example of activation during motor task with the nonimpaired left hand (ML task) of P3. Activation is found in the right precentral gyrus, right putamen, and left cerebellar lobule IV, V, VIII, IX. Color bar represents t-value.

Finger tapping paradigm with the left hand was performed successfully by all pаtients and no mirror movements were observed during the task. Results were pretty consistent in all patients showing activation in their right PMC with different degrees (**Table 2**) and **Figure 3**. Unlike motor task with the impaired hand, additional activation was observed only in regions typically involved with motor processing, like basal ganglia bilaterally or contralateral cerebellum. No extensive activation of other cortical regions was found.

**Table 2:** Activation in the PMC during finger tapping with the nonimpaired hand.

Subject	Lesion	ML: Left motor cortex activation					ML: Right motor cortex activation				
				MNI Coordinates					MNI Coordinates		
		Extent	t-value	x	y	z	Extent	t-value	x	y	z
P1	LMCA infarction	–	–	–	–	–	10,041	6,816	56	−8	46
P2	LMCA infarction	–	–	–	–	–	1,887	3,027	20	−32	74
P3	LMCA infarction	–	–	–	–	–	998	12,310	36	−16	50
P4	LMCA infarction	–	–	–	–	–	3,339	15,151	42	−18	56
P5	LMCA infarction	–	–	–	–	–	600	9,680	44	−22	56
P6	LPVL: small lesion	–	–	–	–	–	3,114	2,517	14	−26	86
P7	LPVL: small lesion	–	–	–	–	–	410	7,889	50	−14	52
P8	LMCD	–	–	–	–	–	1,587	13,104	34	−26	54

ML, motor left; LMCA, left middle cerebral artery; LPVL, left periventricular lesion; LMCD, left malformation of cortical development; MNI, Montreal Neurological Institute.

#### Activation During Language Task

**Figure 4:**
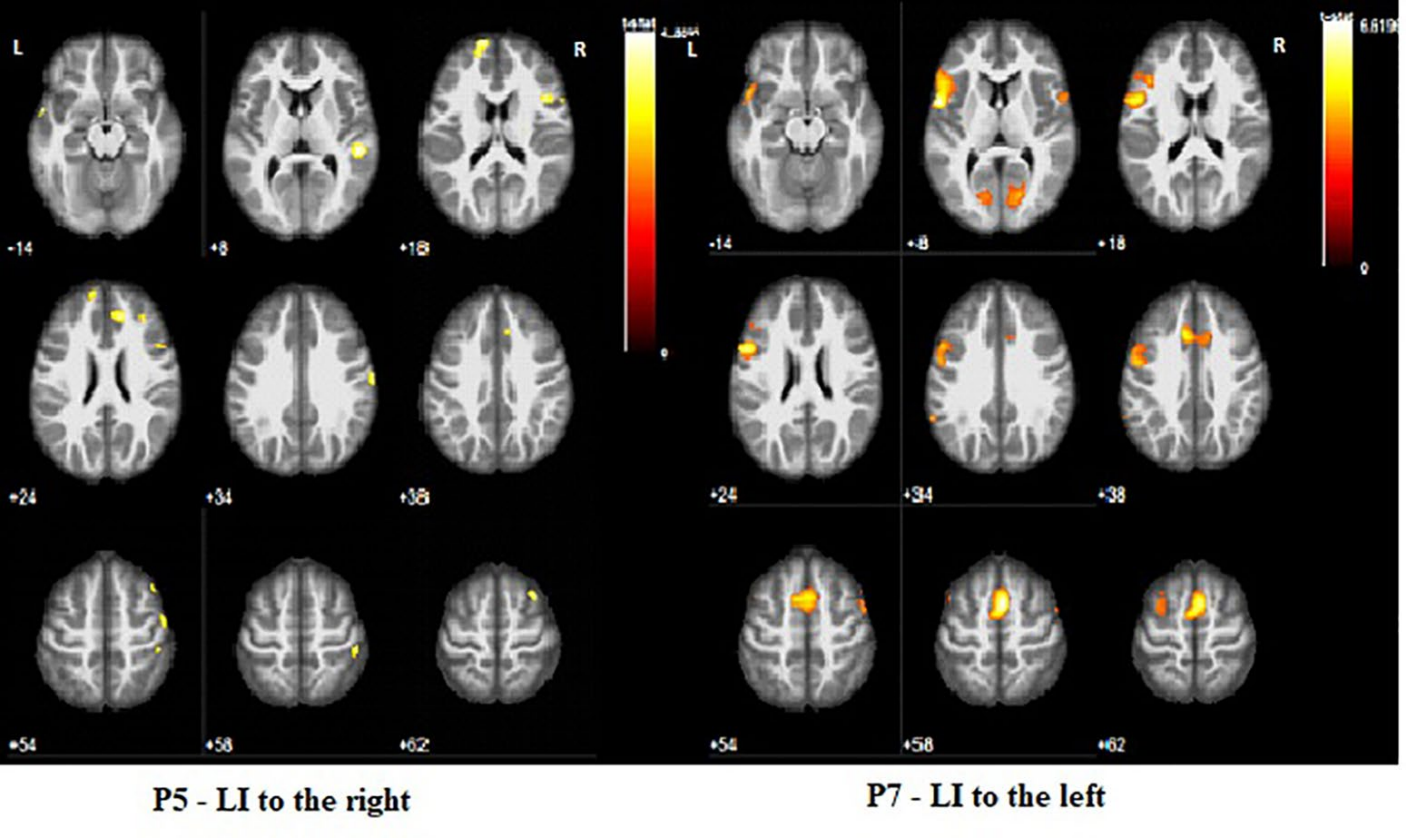
Two types of activation during WG task. None of the patients shown on the figure was found to be dyslexic.

CSL patients showed either right lateralization of cortical activation during the language task (P1, P4, P5), or bilateral one (P2, P3). The MCD (P8) patient showed bilateral activation during the language task. Left-sided predominance (typical for healthy controls) was found in the two PVL patients (P6, P7) (**Table 3** and **Figure 4**).

Dyslexic patients showed various patterns of activation: two with right lateralization, two bilateral, and one left lateralization. Patients who neither had epilepsy nor treatment showed language lateralization shifted to the left hemisphere (P6, P7) or bilateral (P2).

**Table 3:** Activation in right and left hemisphere during the language task (WG).

Subject	Lesion	Dyslexia	Epilepsy	LI (whole brain)	Voxels (right)	Voxels (left)	Clusters (right)	Clusters (left)
P1	LMCA infarction	Yes	Yes	−0.127	23,995	13,986	21	44
P2	LMCA infarction	Yes	No	0.007	38,626	29,979	6	20
P3	LMCA infarction	Yes	Yes	0.082	18,979	16,892	23	44
P4	LMCA infarction	No	Yes	−0.364	36,632	17,031	36	64
P5	LMCA infarction	No	Yes	−0.144	34,436	19,733	12	44
P6	LPVL: small lesion	Yes	No	0.143	55,894	41,593	11	27
P7	LPVL: small lesion	No	No	0.232	44,034	36,726	14	37
P8	LMCD	NA	Yes	0.0426	44,979	36,852	12	20

LMCA, left middle cerebral artery; LPVL, left periventricular lesion; LMCD, left malformation of cortical development; NA, not available; LI, laterality index.

Lateralization to the right hemisphere: 
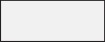

Bilateral representation: 
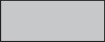

Lateralization to the left hemisphere: 
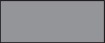

#### Sensory Task—Impaired Hand

Only one patient (P3) could not conduct hand brushing task because she could not endure the full length of the protocol. All patients with PVL and the patient with MCD had left postcentral gyrus activation, as well as the two CSL patients (P4 and P5) in whom the lesion did not encompass the entire left postcentral gyrus. Two other patients with CSL (P1 and P2) showed no activation in the primary sensory cortex; instead there was significant activation of posteriorly or anteriorly located regions. All four patients with CSL showed additional involvement of other areas outside the sensory cortex in both hemispheres. No one showed activation in the contralesional primary sensory cortex during hand brushing task on the impaired hand (SR).

**Figure 5:**
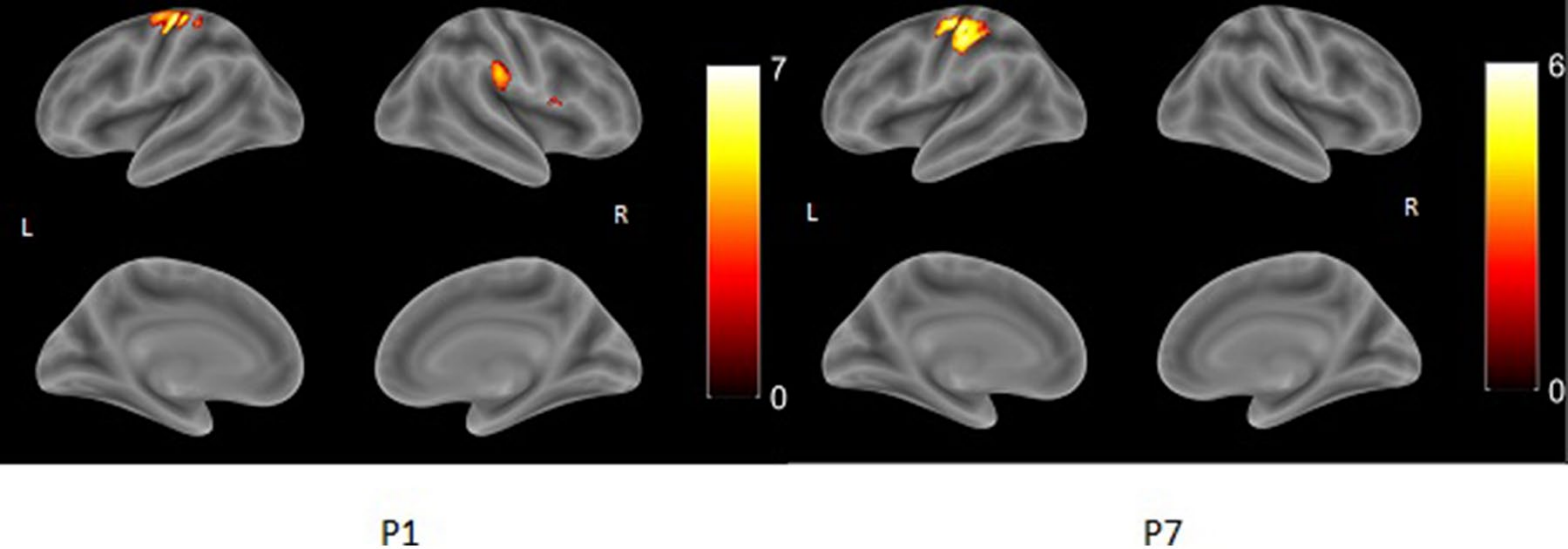
Different patterns of cortical activation during sensory task on impaired right hand (SR). P1 has activation close to left postcentral gyrus, but not located in the gyrus, in contrast to P7 where the activation involves wide zone of the left postcentral gyrus. Color bar represents t-value. Sensory Task—Nonimpaired Left Hand

Comparison with the behavioral data showed some association between the quality of motor function and the area of cortical sensory representation: participants with better motor performance (MACS level I) showing sensory reorganization in the left postcentral gyrus, while participants with worse MACS level (P1 and P2) showing shifting of the activation during SR outside the postcentral gyrus (**Table 4** and **Figure 5**).

**Table 4:** Activation in the primary sensory cortex during SR task.

Subject	Lesion	SR: left sensory cortex activation					SR: right sensory cortex activation				
				MNI coordinates					MNI coordinates		
		Extent	t-value	x	y	z	Extent	t-value	x	y	z
											
P1	LMCA infarction	–	–	–	–	–	–	–	–	–	–
P2	LMCA infarction	–	–	–	–	–	–	–	–	–	–
P3	LMCA infarction	Not conducted				Not conducted					
P4	LMCA infarction	32	5,460,258	−36	−26	70	–	–	–	–	–
P5	LMCA infarction	2,067	781,183	−40	−26	60	–	–	–	–	–
P6	LPVL: small lesion	295	4,890,559	−52	−42	54	–	–	–	–	–
P7	LPVL: small lesion	1,477	6,491,543	−42	−22	58	–	–	–	–	–
P8	LMCD	2,212	1,417,368	−38	−34	60	–	–	–	–	–

SR, sensory right; LMCA, left middle cerebral artery; LPVL, left periventricular lesion; LMCD, left malformation of cortical development; MNI, Montreal Neurological Institute.

Activation outside left postcentral gyrus: 
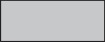

Activation in the left postcentral gyrus: 
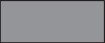

Two patients (one PVL: P6, and one CSL: P1) showed no suprathreshold activation in either left or right primary sensory cortex during hand brushing of the nonimpaired hand. Two CSL patients (P2 and P4) and one MCD (P8) patient had only contralateral activation in right postcentral gyrus; one CSL (P5) and one PVL patient (P7) showed involvement of both primary sensory cortices (**Table 5**).

**Table 5:** Activation in primary sensory cortex during SL task.

Subject	Lesion	SL: left sensory cortex activation					SL: right sensory cortex activation				
				MNI coordinates					MNI coordinates		
		Extent	t-value	x	y	z	Extent	t-value	x	y	z
P1	LMCA infarction	–	–	–	–	–	–	–	–	–	–
P2	LMCA infarction	–	–	–	–	–	94	3,911,956	54	−18	54
P3	LMCA infarction	Not conducted				Not conducted					
P4	LMCA infarction	–	–	–	–	–	1,074	4,129	50	−18	40
P5	LMCA infarction	55	3,710	−62	−16	20	85	3,521	36	−36	66
P6	LPVL: small lesion	–	–	–	–	–	–	–	–	–	–
P7	LPVL: small lesion	1,573	5,771	−64	−26	24	3,626	5,413	46	−26	54
P8	LMCD	–	–	–	–	–	1,160	5,621	28	−32	68

SL, sensory left; LMCA, left middle cerebral artery; LPVL, left periventricular lesion; LMCD, left malformation of cortical development; MNI, Montreal Neurological Institute.

### Discussion

UCP amounts up to 30% of all CPs and is an appealing model for the study of brain plasticity by fMRI because of coexistence of nonprogressive brain lesion in one hemisphere, and normal surrounding tissues in the same one, and non- or less affected opposite hemisphere, which allows the compensation mechanisms for some functions. fMRI shows that the nondominant hemisphere could acquire dominance or codominance in motor, visual, auditory, and language functions after pre/perinatal brain lesions in dominant hemisphere (5).

However, patients with UCP have considerable variability in etiology, time of appearance, size, and location of the lesion, as well as functional capacity, making it difficult to obtain not only large homogeneous patient samples, but also a unified model of brain reorganization. Reported causes of UCP are periventricular white matter lesions, posthemorrhagic porencephalic lesions, infarcts in the middle cerebral artery, and brain malformations (20). Despite the great capacity for plasticity of immature brain and the proposed largely linear relationship between age at brain injury and functional outcome, great variability in outcome from early brain insult is observed, including poor recovery from early prenatal lesions (21).

In our study we applied an individual approach to present the model of reorganization of motor, sensory, and language functions in every patient, and then tried to analyze the established models of brain reorganization in accordance with the etiological type, size and location of the lesion, time of appearance of the lesion, or functional capacity of the patient. Due to the small group of patients, we assume more descriptive approach to our data. Analyzing the reorganization of all three domains within the same patients has not been considered in the previous studies, so we believe that it is the main strength of our study.

### Motor Reorganization

Population studies of children with perinatally acquired unilateral lesions show they have better quality of life than those with bilateral lesions, which is in direct correlation with the better GMFCS (Gross Motor Function Classification System) level (22). Nevertheless those patients usually experience motor, sensory, language, visiospatial, or executive difficulties, which interfere with their everyday life. Children with UCP could never achieve a normal hand function in contrast with language abilities with even minor lesion in the corticospinal tract leading to motor impairment (3).

Our results confirmed the variety of functional motor capacity of patients with pre/perinatally acquired unilateral left hemispheric lesions, demonstrating MACS level varying from I to III.

#### Models of Motor Reorganization

Three models of functional motor reorganization have been found in patients with UCP: only contralesional; only ipsilesional; and bilateral. A recent systematic review showed bilateral activation with stronger contralesional predominance to be the most common model for motor reorganization in UCP (5).

Cao et al. showed bilateral activation during paretic hand movement in all patients, but all of them have cortical lesions (MCDs or CSLs) (23). Similar results in patients with big cortical lesions are reported also by Staudt et al. and Vandermeeren et al. (24, 25). Bilateral activation was reported by Staudt et al. also in patients with small PVL, but in the premotor area (18).

In our study we chose to concentrate on the PMC as site of motor representation. We hypothesize that motor function could be a direct consequence of the number of active neurons left in their original place in the precentral gyrus.

Our study results showed only two out of eight patients had contralesional activation and another two patients bilateral activation. The remaining four patients showed ipsilesional activation which is the rarest model in literature (5). We suppose that methodological and other issues may have an effect on the variability of the models of reorganization, but probably the most important variable is the type and extent of the lesions.

#### Models of Reorganization According to the Type, Location, and Size of the Lesion

Usually patients with lesions involving the left Rolandic area more often had bilateral and/or right predominant activation in M1. Activation of contralesional PMC occurs in patients with severe lesion and absent ipsilesional crossing corticospinal projections, which makes this motor cortex probably the only cortical motor area. Nevertheless, this is also a rare model of reorganization (review **Table 6**). Contralesional activation of motor cortex is suggested to be a result of preservation of the ipsilateral projections from previous stages of development, or to axonal sprouting in “normal” crossed corticospinal axons from the unaffected hemisphere with new collateral branches re-crossing the midline to innervate motor neurons on the paretic side (3).

**Table 6:** Patterns of activation in the PMC during finger tapping task with the impaired hand (MR).

Subject	Lesion	MR—Left motor cortex activation					MR—Right motor cortex activation					LI	MACS
				MNI Coordinates					MNI Coordinates				
		Extent	t-value	x	y	z	Extent	t-value	X	y	z		
P1	LMCA infarction	11,408	5,845,313	−30	−26	54	11,408	55,427	56	−2	24	0	3
P2	LMCA infarction	206	2,538,333	−46	−2	30	2,284	4,886,111	30	−30	68	−0.83	3
P3	LMCA infarction	724	2,143,814	−16	−18	76	1,238	4,193,118	26	−22	72	−0.26	2
P4	LMCA infarction	25,934	880,033	−32	−18	60	None	None	None	None	None	1	1
P5	LMCA infarction	2,103	1,151,858	−32	−22	50	1,626	6,270,804	36	−14	68	0.12	1
P6	LPVL: small lesion	19,322	4,641,092	−32	−32	70	187	2,421,789	16	−34	74	0.82	1
P7	LPVL: small lesion	85	2,796,338	−60	−2	34	None	None	None	None	None	1	1
P8	LMCD	4,900	1,151,858	−32	−22	50	5,197	6,270,804	36	−14	68	−0.03	3

MR, motor right; LMCA, left middle cerebral artery; LPVL, left periventricular lesion; LMCD, left malformation of cortical development; MNI, Montreal Neurological Institute; LI, laterality index.

Ipsilesional activation: 
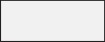

Bilateral activation: 
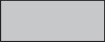

Contralesional activation: 
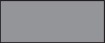

Our patients with CSL with extent of GM and WM injury: two or three showed different models of brain reorganization during motor task with impaired hand; two of five showed contralesional activation of motor cortex; one of five bilateral and two of five ipsilesional. P4 and P5 had large lesions that involve the Rolandic cortex, but not entirely and showed ipsilesional activation during the motor task. We presume that in these two patients the projections from the spared part of the Rolandic cortex played sufficient role in the motor control and didn’t allow the contralesional projections to take over. It is known that the pattern of reorganization varies according to the extent of preservation of the motor area and its connections to the spinal cord (3). However, we could hypothesize that other individual factors like rehabilitation could also play a role in this type of reorganization. In P5, rehabilitation was started early (at 6 months of age) and was conducted daily for years, whereas in P4 no protective factor could be identified, but he was the one with genetically proven thrombophilia (mutation in PAI, ACE, and FV Leiden genes).

Results from patients without cortical involvement (P6 and P7 in our study) are consistent with the findings of Staudt et al. that patients with small PVL show strong ipsilesional activation (18).

#### Models of Reorganization and Time of Brain Injury

Previous studies have hypothesized that timing of the lesion is one of the best predictors for good motor performance and reorganization potential with better functional capacity achieved after early lesion (I, II, and early III trimester) (3). Our study, however, does not show consistency with this theory: P8 having an MCD (timing—II trimester) has severely impaired hand function with MACS level III, while P4 having CSL (timing—late III trimester) has almost normal hand function with MACS level I. However, our study population included only one patient with brain lesion occurring earlier than III trimester, like MCD, which doesn’t allow definite conclusion on this matter.

#### Models of Reorganization and Motor Performance

Our results showed association between left-sided lateralization of the activation in M1 and better hand performance, which is in support of the thesis that normal or near-to-normal hand function seems possible only with preserved crossed corticospinal projections from the contralateral hemisphere. Similar results have been found in the TMS study of Holmström et al. with better performance on the Box and Blocks test and AHA (Assisting Hand Assessment) of children with ipsilesional motor projections, as well as in the study of Jang et al. (26, 27). Fiori et al. in a case report also discussed that it was not very likely for the intact contralesional hemisphere alone to be sufficient for a normal function of the ipsilateral hand in the presence of an early unilateral lesion in the opposite hemisphere (28).

Mackey et al. found correlation between preserved ipsilesional motor control and hand motor function and suggested it as a result of intact intracortical and interhemispheric inhibition (29). The other explanation of this correlation is the “crowding” theory and the effect of representation of motor function for both hands in one cortical region with impossibility of one motor cortex to be “enough” for both hands (27).

Based on our results we could also conclude that patients with UCP demonstrating ipsilesional activation of PMC during motor task have better motor functional capacity.

### Sensory Reorganization

Unlike other studies none of our patients experienced sensory deficits, even those with cortical lesions involving big part of the postcentral gyrus.

Despite the variability in age, gender, clinical characteristics, level of sensory deficits, and type of lesion, patients with UCP demonstrated predominant compensation through ipsilesional reorganization of the sensory function (30, 31). Recent systematic review revealed that almost all patients showed activation only in the ipsilesional hemisphere during sensory tasks and our results were in consistency with previously reported data (31–35). We found that reorganization in the sensory system occurred ipsilateral to the lesion independently on either its type or extent, or type of motor reorganization. Patients with damaged sensory cortex typically activate the intact portions of the postcentral gyrus with a somewhat more variable topography (36). Functionally, many of these patients show severe somatosensory deficits, which sometimes contrast with relatively spared motor abilities (36). However no one of our patients was found to have sensory deficits, despite the wide destruction of the primary sensory cortex in some patients.

It is interesting to discuss the possible relationship between sensory and motor functions and their cortical representations in patients with UCP.

According to our results, representation of primary motor and primary sensory function in different hemispheres is associated with worse hand function. This dissociation of lateralization is determined only by the type of motor representation, because sensory reorganization was found to be always ipsilesional. The possible explanation of this result has been commented in the discussion of motor reorganization, but other explanation of impaired motor function might be the dissociation of sensory input and motor output to different hemispheres. This has been suggested by several authors (30, 34). Bigger destruction of the primary sensory cortex with shifting of the sensory function to the neighboring cortical areas also leads to worsening of the motor function (P1 and P2). Both concepts (motor and sensory presentation in different hemispheres, and atypical ipsilesional sensory representation) may hamper the sensorimotor integration, which is important for skillful hand movements.

Considering the few number of studies and the small groups of patients (30–36), further research on the reorganization of the sensory system in UCP, especially in combination with the reorganization of the motor and language systems, will contribute to better understanding of brain neuroplasticity.

### Language Reorganization

Children with pre- or perinatal brain injury of the dominant hemisphere could acquire age-appropriate language, in contrast to the aphasia following similar lesions in adulthood (37, 38). Recent studies support the hypothesis of the “dormant circuitry” available for language function that is inhibited in the nondominant hemisphere of healthy children, but which may be activated when primary regions in the dominant hemisphere become unavailable to exert inhibition (39–42). A shift of language production to the right hemisphere (zones that are homotopic to the original left language zones) has been registered in most children with congenital left hemispheric lesions by dichotic listening tests (43–45) and by fMRI (46–52). However, left hemisphere lesion does not obligatorily induce a shift of language representation to the right hemisphere (48, 50).

Several models of language reorganization in patients with right-sided UCP and left-sided brain lesions are described: bilateral activation with either ipsilesional or contralesional dominance; only ipsilesional activation; only contralesional activation. Bilateral activation with contralesional dominance prevails in more than a half of the investigated patients (5). Our results are in concordance with these data: three out of eight patients demonstrated bilateral activation; two of them suffering from large left MCA infarction and one with schizencephaly; three other showed contralesional (right-sided) activation, all of them with CSL and also large injury (extent of GM and WM lesion ≥2); and only 2 patients with small PVL had ipsilesional activation.

Patients with left MCA infarction, i.e., CSL, demonstrated contralesional or bilateral activation and none of them had ipsilesional activation which is probably the result of the destruction of their primary language cortex, while patients with small PVL and preserved language cortex had ipsilesional activation. Therefore the dominant contralesional activation occurred only in patients with large CSL (extent of injury ≥2), and not with PVL, which supported the thesis that both the site and extent of a left hemispheric lesion determine the capacity for reorganization. Knecht and Lidzba and Lidzba et al., like us, reported a prevalence of greater right hemispheric language dominance in cortical lesions compared to PVL (53, 54).

Raja Beharelle et al. suggested that language reorganization depended more on the type rather than the size of lesion (50). However, LI values were inversely correlated with severity of the lesion according to Chilosi et al.: in cortical and subcortical, but not in PVLs right hemisphere language dominance is significantly associated with more severe brain damage, and our results supported this conclusion (42).

According to some authors, lateralization of language areas should be regarded differentially according to the cortical regions: UCP patients with better language outcome show a functional organization for language that favors left over right activity in frontal brain regions and a bilateral pattern of activity in right and left temporal-parietal regions (50).

The association between language and intellect is also discussed. Some researchers consider that the greater the shift to the right of language functions, the lower the cognitive and expressive language scores (40, 42). However, we could not support these statements, because two of our patients with right-sided contralesional language activation had high IQ, 89 and 90, respectively, in contrast with the other two patients with bilateral activation with IQ, respectively, 50 and 60. According to our results the type of language reorganization does not predict the language outcome. Our study confirmed the thesis that atypical language lateralization (in terms of LIs) is not necessarily associated with impaired performance during experimental tasks (55–57).

#### Reorganization According to Dyslexia

The children with CP had poorer phonological processing abilities than controlled typical children (58, 59), and these abilities correlate with their reading skills (60, 61). Several studies point out that reading recognition and reading comprehension abilities are lower than verbal intelligence in patients with cerebral palsy, although there were some inconsistencies in the findings (62, 63). In accordance with these data, five out of seven of our patients had dyslexia and three of them were with normal intelligence (IQ above 70).

Language activation in adults with isolated dyslexia is slightly right lateralized, in contrast with typical readers with left-lateralized activation. This suggests that the activation in the right hemisphere in isolated dyslexic individuals is likely to be the cause rather than the consequence of reading impairment (64). These speculations may be inferred to UCP patients with dyslexia, although the effect of the structural damage should not be underestimated. Accordingly, four out of five patients with dyslexia in our study had right hemispheric activation with or without left hemispheric activation during silent word generation task.

Four out of five patients with CSL and one out of two patients with PVL had dyslexia, i.e., it could be suggested that left hemispheric lesion, especially including left frontal cortex could result in dyslexia. The left inferior frontal gyrus is associated not only with articulation but also is involved in phonological processing (65). Activation in this area is positively correlated with reading ability (65).

Dyslexia correlates to some extent with motor reorganization and performance in our patients as three out of five dyslexic patients had worse MACS grades (grade 2 and 3) and contralesional or bilateral activation during the motor task, while the two patients without dyslexia had better MACS grades (grade 1) and ipsilesional motor activation.

These results require further studies to clarify the relationship between dyslexia and the type and size of the lesion in left hemisphere.

#### Limitations and Factors Influencing the Results

The strongest limitation of the language fMRI task was the impossibility to evaluate the exact execution of the task by the participants inside the MRI, although all the participants were asked to reproduce verbally the task after the experiment. Silent, but not vocal, word generation is really important to the experiment in order to avoid activation in motor areas involved in language production.

Many factors could influence the task performances, either related or unrelated directly to UCP. In terms of age, there is evidence in previous studies that left lateralization for language production gets stronger with age (66–68). This stronger shift to the left hemisphere occurs in healthy subjects in late childhood and adolescence and is independent of the region of interest used for calculation of LI (whole brain, prefrontal cortex, frontotemporal regions) (68). Our study population, however, contains patients between age 11 and 29 and these age effects should be minimal or finalized.

Epilepsy is a common comorbidity in patients with CP, as well as in our patient group (five of eight patients have epilepsy and are under medication). There are evidences that both epileptic activity and medication (especially carbamazepine) could influence cognition and cognitive and language representation in the brain (69–71). All five patients with epilepsy in our study had atypical language representation (bilateral or contralesional—in the right hemisphere), so it could be speculated that factors playing a role in “shifting” of verbal production in the right hemisphere could be a result not only from the lesion itself, but also from other factors like epileptiform activity or antiepileptic drugs. The possible effects of epileptic activity on language representation was discussed by Lidzba et al., and a suggestion was made that evaluation of language production in nonepileptogenic lesions is somehow more reliable due to the lack of confounding effect of epileptic activity (54). In larger sample study antiepileptic drugs and epileptiform activity could be evaluated as predictive or significant factors for language representation.

## Conclusion

Despite the limitations of the study (small sample, different type of brain lesions, some confounding factors), several conclusions could be made:

- Patients with small PVLs have ipsilesional representation of primary motor, sensory, and word generation function. This, however, does not strictly correlate with better outcome, especially in terms of language and cognition—one of the patients has borderline IQ score and the other one is dyslexic, although with normal intelligence, but both had very good motor capacity and no sensory deficit.
- Patients with lesions involving left CSL regions show various models of reorganization in all three modalities (ipsilesional, contralesional, and bilateral) and different clinical outcomes that seem to be impossible for prediction. Anyway, there is a tendency of larger lesion being associated more frequently with motor and language shift to the contralesional hemisphere, and atypical location of primary sensory cortex. Patients with UCP who demonstrate ipsilesional motor cortical activation have better motor functional capacity.

As this is a pilot study with only eight patients, the conclusions made are exploratory. Much larger sample and additional correlation with morphological data (volumetry, morphometry, tractography) is needed for determination of possible risk or protective factors that could play a role in the complex process of brain plasticity. Despite the mentioned limitations of the study, it is the first one that explores brain plasticity in three modalities at the same time with comparison to anatomical and clinical data.

## Data Availability Statement

All datasets generated for this study are included in the article.

## Ethics Statement

The studies involving human participants were reviewed and approved by Ethical committee of Medical University–Plovdiv. Written informed consent to participate in this study was provided by the participants’ legal guardian/next of kin.

## Author Contributions

Conceptualization: KG, IP, and II. Methodology: KG, IP, and II. Validation and formal analysis: KG, IP, and II. Investigation: KG, IP, II, ET, AP, and KV. Resources: KG, IP, II, ET, AP, and KV. Data curation: KG, IP, II, ET, AP, and KV. Original draft preparation: KG, IP, and II. Writing—review and editing: KG, IP, II, ET, AP, and KV. Visualization: IP, KG, and II. Project administration: KG, II, and KV.

## Conflict of Interest

The authors declare that the research was conducted in the absence of any commercial or financial relationships that could be construed as a potential conflict of interest.

The handling editor declared a shared affiliation, though no other collaborations, with several of the authors KG, IP, ET, AP, KV, and II at time of review.
